# Special Issue “Micro/Nano Emulsions: Smart Colloids for Multiple Applications”

**DOI:** 10.3390/nano12213734

**Published:** 2022-10-24

**Authors:** Ruggero Angelico

**Affiliations:** Department of Agricultural, Environmental and Food Sciences (DIAAA), University of Molise, Via De Sanctis, 86100 Campobasso (CB), Italy; angelico@unimol.it

Microemulsions are known as thermodynamically stable nanodispersions driven by spontaneous emulsification and are commonly prepared as transparent mixtures composed of oil, water, a surfactant, and a cosurfactant. The latter are two key components implied in the stabilization process of the microemulsions due to a critical reduction in the interfacial tension between immiscible phases and an increase in the surface area of the internal droplets. These versatile colloidal systems favor the generation of various microstructures and the encapsulation of various drug molecules into either the lipophilic or the hydrophilic phase, resulting in droplets with hydrodynamic diameters up to 100 nm. Depending on the components’ proportions, the microstructure of microemulsions ranges from tiny water droplets dispersed in the oil phase (W/O microemulsion) to oil droplets dispersed in the aqueous phase (O/W microemulsions). In other regimes of composition, bi-continuous structures consisting of interdispersed oil and water nanodomains separated by flexible surfactant monolayers can be obtained as well. The applications of microemulsions include a wide span of areas such as food science, detergents, lubricants, coatings, antibacterial and pesticides, cosmetics, drug delivery, nanoparticle synthesis, biotechnology, isolation and extraction, chemical reactors, templates for gelification, and even restoration of works of art.

Nanoemulsions (i.e., submicrometer-sized emulsions with droplet size ranging from 50 to 200 nm) hold considerable potential in a variety of applications, e.g., in the pharmaceutical field, functional food design, cosmetics, agrochemicals, and fuels. The success of nanoemulsions derives from the small droplet size, which in turn confers high kinetic stability to the colloidal system. Nanoemulsions have many incomparable advantages over other nanocarriers, such as (i) a simple preparation process, no special equipment, and easy operation; (ii) a wide surface area provided by the submicrometer-sized droplets, uniform droplet dispersion, and high stability; and (iii) improvement in bioactive solubility and bioavailability. 

A substantial number of papers were submitted to this Special Issue of *Nanomaterials*, which has been inspired by the two classes of colloidal systems briefly outlined. Hence, after a thorough peer review process, a total of nine papers were collected: five as research articles and four as reviews. As briefly summarized below, these papers cover a wide range of scientific results addressing advanced applications in the field of the colloidal science of micro- and nanoemulsion systems. 

Pham-Van et al. [1] numerically simulated the self-assembly of 2D clusters of colloidal particles assisted by the evaporation of an emulsion stabilized by nanoparticles (Pickering emulsions). The authors argued that the structure of small clusters was dictated by the minimization of the second moment of the mass distribution (M_2_). The 2D configurations were found to favor geometric figures known as polyiamonds (planar figures formed by joining congruent equilateral triangles edge to edge). The number of ways to generate particle packing was associated with the probability of a particular configuration. The structure of small clusters was insensitive to the detailed shape of the inter-particle potential, thus demonstrating universal features of symmetry. 

Shakeel and co-workers [2] reported the first attempt to stabilize oil/water (O/W) emulsions through both interfacial and bulk modification in the same system. This synergistic stabilization was achieved by the adsorption of silica nanoparticles at the oil–water interface and the incorporation of polymeric gelators in both phases. The authors compared the rheological properties of different formulations including hydrogel, organogel, conventional bigel (structured oil and water phases without nanoparticles), hydrogel Pickering emulsion (unstructured oil and structured water phases with nanoparticles), organogel Pickering emulsion (structured oil and unstructured water phases with nanoparticles), and nanoparticle-based bigels (structured oil and water phases with nanoparticles). The results showed the synergistic enhancement in the complex modulus of nanoparticle-based bigels as compared to conventional systems, a peculiar effect that was attributed to the bulk stabilization of the system with polymeric gelators and the interfacial stabilization with the help of nanoparticles. 

Caianiello et al. [3] reported the physicochemical properties of a novel nanoemulsion formulated by phospholipids (PLs) able to favor inverse curvatures, namely water/oil (W/O) emulsion systems. The authors were inspired by the LPs composition of the inner leaflet of the Gram-negative bacteria’s outer membrane, which is highly rich in phosphoethanolamines (PE) and phosphatidylglycerols (PG). Therefore, the authors tested the effect of a PE/PG mixture (7/3 mol ratio) on the stability of W/O emulsions formulated with 0.5 vol % of water and two continuous phases, i.e., dodecane and squalene. To prevent droplet coalescence, small amounts of a glycosylated surfactant were added to the original phospholipid mixture with the aim of providing a compact superficial coating for the water droplets. 

The authors showed that while an emulsion stabilizer was necessary to obtain relatively stable PLs water/dodecane nanoemulsions, no additional component was required when squalene was used as the oil phase. Their study emphasised the importance of the bioinspired design as a strategy to achieve stable biocompatible nanostructured formulations, thus fostering their potential biotechnological applications. 

Esposito et al. [4] presented an experimental investigation of the pseudo-phase diagrams of emulsions formed by water and, alternatively, a linear or a branched mineral oil stabilized by mixtures of glycosylated non-ionic surfactants, characterized by the same hydrophobic tail, while the hydrophilic heads were different in size. The electron paramagnetic resonance (EPR) spectroscopy was found to be very useful for investigating the surfactant microscopic organization at the droplet interface. The results confirmed the ability of glycosurfactants to stabilize O/W emulsions in a whole range of surfactant mixture composition. It has been shown that the surface of the O/W droplets, characterized by a positive curvature, were able to accommodate both types of headgroups without being affected by the arrangement of the surfactant tails. However, a different result was obtained for the inverse type of W/O emulsions for which it was found that the negative curvature of the reversed droplets was more stable if the composition of the interface was mainly constituted by the more hydrophobic surfactant. As a whole, the authors reported a clear relation between the surfactant layer microstructure and the emulsion structure at the nanoscopic scale, finally determining the system’s macroscopic stability.

Chen et al. [5], in their paper, shed light on the effects of chymosin on the physicochemical and hydrolysis characteristics of casein micelles as well as of individual milk proteins. The destabilization and coagulation of casein micelles induced by chymosin play a specific function during the bioactive reactions in the cheese-making process. Results acquired by mass spectrometry and SDS-PAGE techniques showed that chymosin was indeed responsible for the reduction in the molecular weights of hydrolyzed casein proteins, which allows further knowledge to be gained on the complex mechanism of the cheese-making process. 

Caputo et al. [6] provided a detailed review of the available scientific results achieved in the field of Warm Mix Asphalt (WMA) technology. Differently from the conventional Hot Mix Asphalt (HMA) methods, the WMA technology reduces the compaction temperatures of asphalt mixtures by 20–40 °C, leading to a significant reduction in environmental pollution as well as production costs for the road paving industry. This review systematically exposed various procedures indicated for WMA techniques, such as the addition of wax and chemical additives and the use of foaming processes, including the mechanisms by which they act to impart the desired characteristics and improve the durability of the mix. Moreover, the authors in their review mentioned other innovative techniques, such as the use of stabilized emulsions to produce WMA. Indeed, the water/bitumen emulsions stabilized by suitable emulsifiers have the advantage that after the pre-coating of the aggregates with the bituminous emulsions, the water is removed by evaporation and the residual surfactant favors the adhesion of the bitumen onto the inert. 

Mustafa et al. [7] in their review reported an updated state of the art on the potential for the application of nanoemulsion technology in different agricultural sectors, such as nano-chemicals for agriculture, nano-fertilizers, or encapsulating materials. Specifically, they discussed the preparation protocols, physicochemical and biological characterizations, and technology of nanoemulsion-based pesticide formulation for agricultural use. For example, both low-energy (together with its solvent-free variant) and high-energy methods of preparation applied to pesticide-based nanoemulsion formulations have been clearly illustrated and analyzed by the authors. Studies of the use of nanoemulsions for pesticide delivery in both in-vitro and in-vivo applications have also been reviewed, including a discussion on environmental risk assessment. 

In their review, Tartaro et al. [8] examined and summarized the physicochemical properties exhibited by complex fluids falling in the definition of microemulsions, such as large surface area, very low interfacial tension, and high macroscopic stability, which are widely exploited in the preparation of multiple types of nanomaterials. The authors highlighted the considerable efforts made by the scientific community to rationalize and predict the relationship between the composition of the microemulsion and its microstructure. 

In particular, they discussed the concept of hydrophilic–lipophilic difference (HLD), which represents a powerful model capable of handling in a single equation the chemical nature of oil and surfactant, as well as temperature and salinity. Indeed, the empirical relationship applied for the determination of HLD allows a formulator to create stable emulsions where the dispersion phase and continuous phase are predictable (O/W or W/O). The authors introduced also the net average curvature (NAC) model, which provides a framework where either water or oil, or both, can be the continuous phase of a ternary surfactant–oil–water system, allowing for the coexistence of positive and negative curvatures. Furthermore, the review includes a schematic discussion of microemulsions in terms of phase diagrams and how they change as a function of temperature and compositions. 

Finally, Talianu et al. [9] reviewed research papers dealing with the application of biocompatible microemulsions entrapping active pharmaceutical ingredients as topical treatments aimed at promoting the healing process in dermatological diseases. These colloidal systems exploit the ability to overcome the skin barrier of the stratum corneum by delivering lipophilic drugs with anti-acne activity in the affected site. The authors summarized the main criteria followed by the design of the microemulsion, giving rise to their optimized compositions, capable of promoting a more efficient delivery than conventional formulations. The review represented a useful reference on the state of the art of microemulsion formulations proposed to improve the skin bioavailability of both hydrophilic and lipophilic anti-acne compounds. 

As a final remark, I trust that the papers collected in this Special Issue draw attention to the current relevant topics in research related to the molecular self-assembly of liquid nanomaterials such as micro- and nanoemulsions, and introduce readers to advanced applications in this field.
